# Supplementary material for: Role of chemisorbing species in growth at liquid metal-electrolyte interfaces revealed by in situ X-ray scattering
Source: Nat Commun. 2022 Sep 15;13:5421. doi: 10.1038/s41467-022-32932-7 (PMC9477831; doi:10.1038/s41467-022-32932-7)
Supplement: Supplementary file 1 — Supplementary information: Role of chemisorbing species in growth at liquid metal-electrolyte interfaces as revealed by in situ X-ray scattering [file 41467_2022_32932_MOESM1_ESM.pdf]

Supplementary information

Role of chemisorbing species in growth at liquid metal-electrolyte interfaces as revealed by in situ X-ray scattering

Andrea Sartori[1,2], Rajendra P. Giri[1], Hiromasa Fujii[1,3], Svenja C. Hövelmann[1,4], Jonas E. Warias[1], Philipp Jordt[1], Chen Shen[4], Bridget M. Murphy[1,5 *], Olaf M. Magnussen[1,5 *]

[1] Institute for Experimental and Applied Physics, Kiel University, 24118 Kiel, Germany

[2] Present Address: European Synchrotron Radiation Facility, 38000 Grenoble, France

[3] Present Address: Mitsubishi Electric Corporation, Advanced Technology R&D Center, 8-1-1 Tsukaguchi-Honmachi, Amagasaki 661-8661, Japan[4] Deutsches Elektronen-Synchrotron DESY, Notkestrasse 85, 22607 Hamburg, Germany

[5] Ruprecht-Haensel Laboratory, Kiel University, 24118 Kiel, Germany

*email: magnussen@physik.uni.kiel.de; murphy@physik.uni.kiel.de

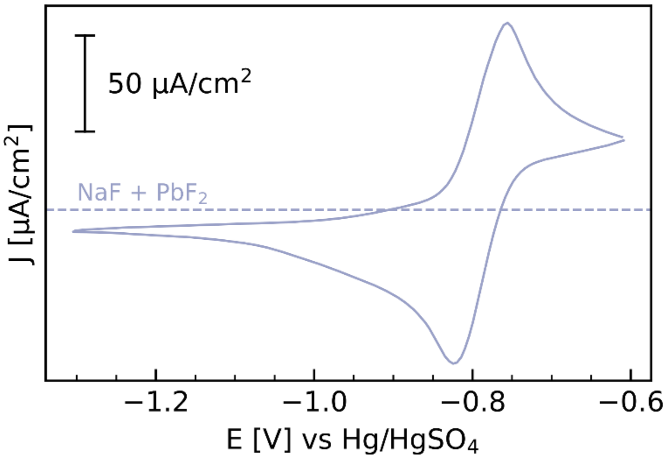

Supplementary Fig. 1 | Cyclic Voltammetry of Hg in NaF + PbF_2 after positive bias. Cyclic voltammogram of Hg in 0.01 M NaF + 0.25 mM PbF_2 (blue solid line), for clarity, current density J = 0 indicated by blue dashed horizontal line. The CV was measured at 20 mV/s after keeping the potential for periods of 60 min at potentials positive of -0.8 V.

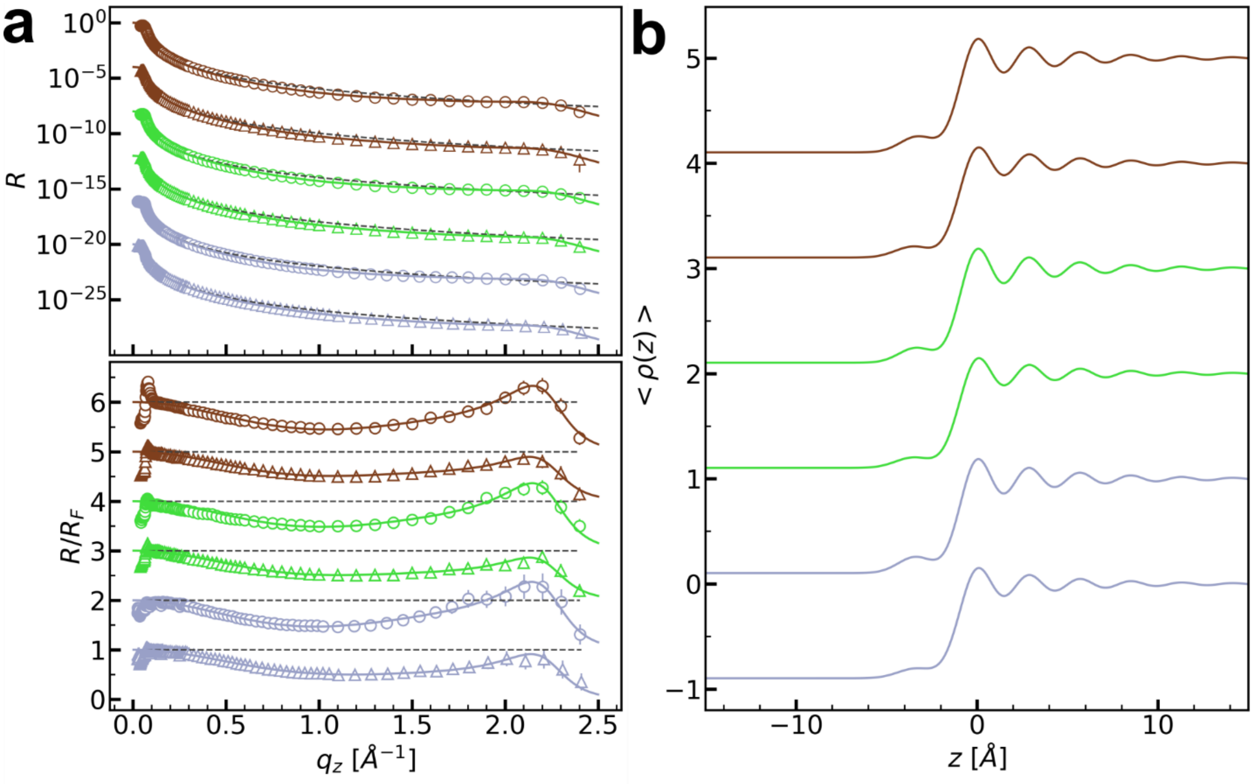

Supplementary Fig. 2 | X-ray Reflectivity of Hg in lead free solutions. a Reflectivity profiles (top panel) and reflectivity normalized by the Fresnel reflectivity $R_F(\mathbf{q_z})$ (bottom panel) of the liquid Hg electrode in pure 0.01 M NaBr (brown), 0.01 M NaCl (green) and, 0.01 M NaF (blue) solution. Triangles indicate XRR curves measured at -1.20 V, circles XRR curves measured at -0.60 V. top: The grey dashed lines indicate the Fresnel reflectivity $R_F(q_z)$, solid lines correspond to the best fit of the XRR data. For clarity, the individual profiles are offset with respect to each other. The error bars in **a** represent the instrumental errors, resulting from the counting statistics of signal and background. **b** The electron density profiles corresponding to the best fits (detailed structural parameters are given in Supplementary Table 1).

Supplementary Table 1 | Structural data on the Hg-electrolyte interface structure in Pb-free solutions.

System	Parameter	-1.20 V	-0.60 V
Common values	d [Å]	2.76	2.76
	σ_b [Å]	0.48	0.48
	z_{H_2O}[Å]	1.50	1.50
0.01 M NaBr	σ_i [Å]	1.01±0.01	0.97±0.01
	ρ_{ad}	0.27±0.01	0.37±0.01
	z_{ad}	3.51±0.03	3.25±0.04
0.01 M NaCl	σ_i [Å]	1.01±0.01	0.96±0.01
	ρ_{ad}	0.26±0.01	0.35±0.01
	z_{ad}	3.48±0.05	3.34±0.04
0.01 M NaF	σ_i [Å]	1.01±0.01	0.96±0.01
	ρ_{ad}	0.24±0.01	0.38±0.01
	z_{ad}	3.35±0.05	3.38±0.03

The standard errors of the best-fit parameters were calculated by the covariance matrix method.

Supplementary Methods 1: Fitting process and model

In order to fit the experimental XRR data the distorted crystal model (DCM) was used. The DCM described the near surface region of a liquid metal by an infinite stack of layers that continuously broaden with increasing distance from the surface. Based on this model, the electron density profile of a simple Hg-electrolyte interface is described by the following equations:

$$\rho_{DCM}(z) = F_{Hg} \otimes \left(\sum_{n=0}^{\infty} \left(\frac{d\, \rho_{Hg}}{\sigma_n \sqrt{2\pi}} \exp\left(-\frac{(z-nd)^2}{2\sigma_n^2} \right) \right) + \frac{\rho_{ad}}{\sigma_{ad}\sqrt{2\pi}} \exp\left(-\frac{(z-z_{ad})^2}{2\,\sigma_{ad}^2} \right) \right) \tag{1}$$

$$\text{With } \sigma_n = \sqrt{\sigma_i^2 + n\,\sigma_b^2} \tag{2}$$

$$\rho_{electrolyte}(z) = F_{H_2O} \otimes \left(\frac{\rho_{H_2O}}{2} \left(1 - \mathrm{erf}\left(\frac{z - z_{H_2O}}{\sigma_{H_2O}} \right) \right) \right) \tag{3}$$

$$\frac{\langle \rho(z) \rangle}{\rho_\infty} = \frac{\rho_{DCM}(z) + \rho_{electrolyte}(z)}{\rho_{Hg}} \tag{4}$$

Here, d describes the interatomic distance between the atomic layers of mercury, σ_i describes the intrinsic width common to all layers, which corresponds to the roughness of the Hg surface layer (n = 0), and σ_b determines the rate by which σ_n increases with n. The second term of the eq. 1 describes an additional Hg adlayer of lower density, which is a typical feature of Hg-vapor and Hg-liquid interfaces[1–4]. ρ_{Hg} and ρ_{H_2O} are the electron density of mercury and water respectively. F_{Hg} and F_{H_2O} are the Fourier transform of the atomic form factor of Hg and H_2O. Using the form factor of water for the description of the electrolyte is justified by the low concentration of the employed solutions.

In the fits of the reflectivity curves in the de-amalgamation potential regime, adlayers of Pb halide compounds were included in the electron density profiles. These typically consisted of several atomic layers of different ionic compounds (Pb, halides, OH) that were placed at defined distances. The electron density contribution of each of these atomic layers is given by:

$$\rho_n(z) = F_n \otimes \left(\frac{\theta}{\sigma_n \sqrt{2\pi}} \exp\left(-\frac{(z - z_n)^2}{2\, \sigma_n^2} \right) \right) \tag{5}$$

For the XRR data obtained in Br- or Cl-containing solution, the distances between the positions of the atomic layers z_n and the type of the atomic species (defined by the Fourier transform of the atomic form factor F_n) were fixed to those in the corresponding bulk compounds described in the main manuscript. The layer widths σ_n were identical for all atomic layers and fixed to that of the Hg surface layer (i.e., $\sigma_n = \sigma_i$). Likewise, all layers were scaled by the same coverage factor θ. In the F-containing solution a more general approach was applied. Here, a sequence of up to three Gaussian with independently fitted densities ρ_n, widths σ_n, and positions z_n, was used (n = 1, 2, 3). The sum of all contribution

$$\frac{\langle \rho(z) \rangle}{\rho_\infty} = \frac{1}{\rho_{Hg}} \left(\rho_{DCM}(z) + \rho_{electrolyte}(z) + \sum_n \rho_n(z) \right) \tag{7}$$

was then inserted in the master formula:

$$\frac{R(q_z)}{R_F(q_z)} = \left| \frac{1}{\rho_\infty} \int \frac{d\langle \rho(z) \rangle}{dz}\, e^{iq_z z}\, dz \right|^2 \tag{8}$$

where $R_F(q_z)$ is the Fresnel reflectivity of an ideally sharp interface and $\mathbf{q_z}$ the scattering vector in surface normal direction. This expression was used to fit the experimental data $R(q_z)$.

The fit model was implemented in a self-written Python code. The first stage of the fit procedure was to fit all the reflectivity of Hg in NaX (X=Br, Cl, F) where the interface is pristine. The average bulk parameters of mercury (σ_b , d) were then used to fit all the rest of the data presented in the manuscript. The first step of each fit was to set a wide range of boundaries for each parameter, by using a global optimization. The obtained values were then employed as starting point for a second fit with a local optimization algorithm. The error function describing the electrolyte solution has only a small effect on the fit of the Hg-electrolyte interface in the absence of a layer. Therefore, also the position of the error function was fixed to the average value.

The quality of the fit was judged from the χ^2 value of the fit. It was considered excellent with a value ≤ 0.05 and acceptable up to 1.5. If χ^2 exceeded this threshold, the initial values, parameter's boundaries, constrains and/or model were re-considered.

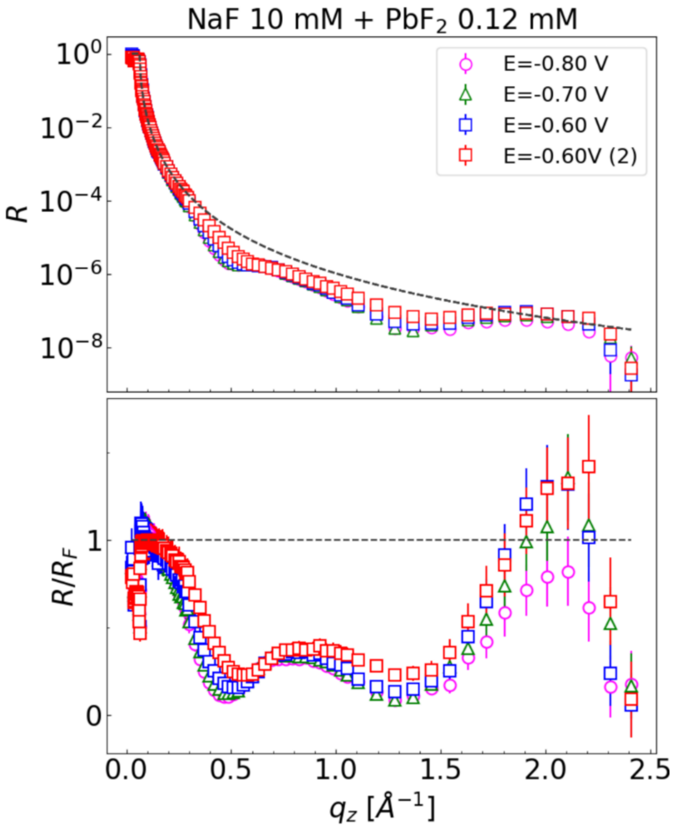

Supplementary Fig. 3 | Temporal changes in the XRR obtained in F-containing electrolyte. Series of XRR, recorded subsequently on Hg in 0.01 M NaF + 0.12 mM PbF_2 at -0.80 V, -0.70 V, -0.60 V, and again at -0.60 V. Prior to these measurements, the sample had been kept at -1.20 V for 15 minutes. The data indicate that the adlayer is not stable but slowly changes with time, even if the sample is kept at the same potential. The error bars represent the instrumental errors, resulting from the counting statistics of signal and background.

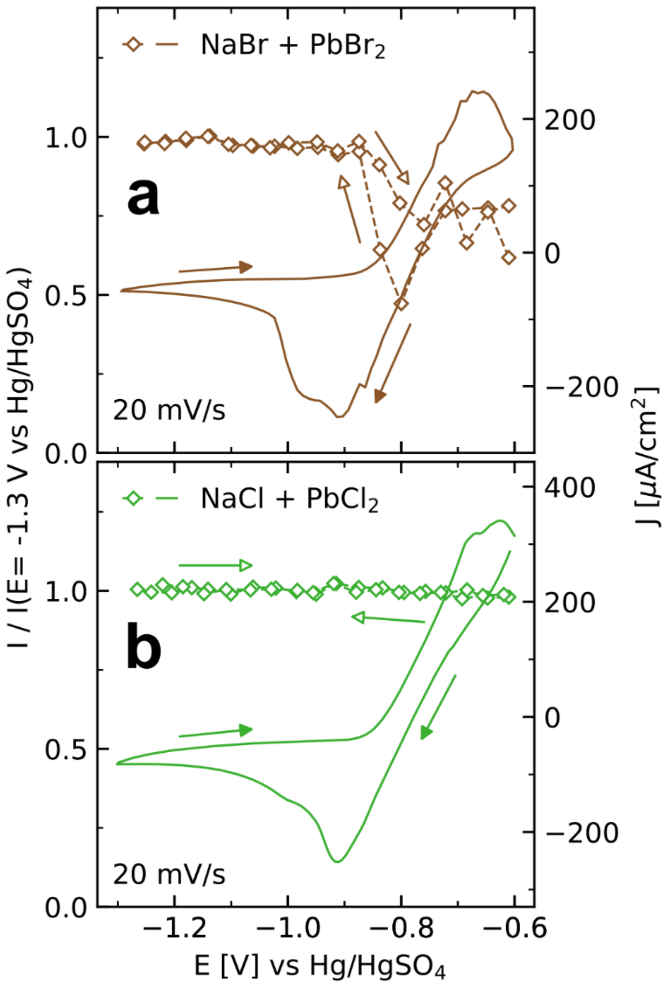

Supplementary Fig. 4 | Potentiodynamic X-ray experiment with freshly prepared samples. X-ray intensity (open symbols) at **a** $\mathbf{q_z}$ = 0.60 $Å^{-1}$ and **b** $\mathbf{q_z}$ = 0.45 $Å^{-1}$, recorded during cyclic voltammograms (solid lines) of Hg at 20 mV/s in **a** 0.01 M NaBr + 0.25 mM $PbBr_2$, **b** 0.01 M NaCl + 0.25 mM $PbCl_2$, recorder with a freshly prepared sample, i.e. without keeping the sample for a long period of time at potentials more positive than E = - 0.80 V. Arrows indicate the directions of the potential sweeps.

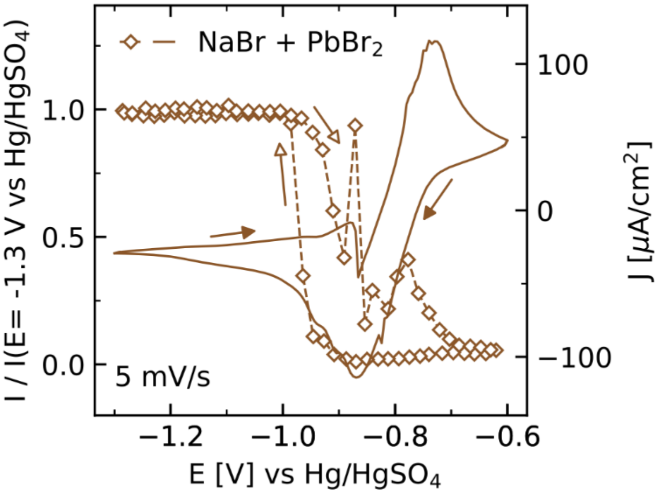

Supplementary Fig. 5 | Potentiodynamic X-ray experiment showing strong intensity fluctuations in the transition region. X-ray intensity (open symbols) at $\mathbf{q_z}$ = 0.45 $Å^{-1}$, recorded during cyclic voltammogram (solid lines) of Hg at 5 mV/s in 0.01 M NaBr + 0.25 mM $PbBr_2$. Arrows indicate the directions of the potential sweeps.

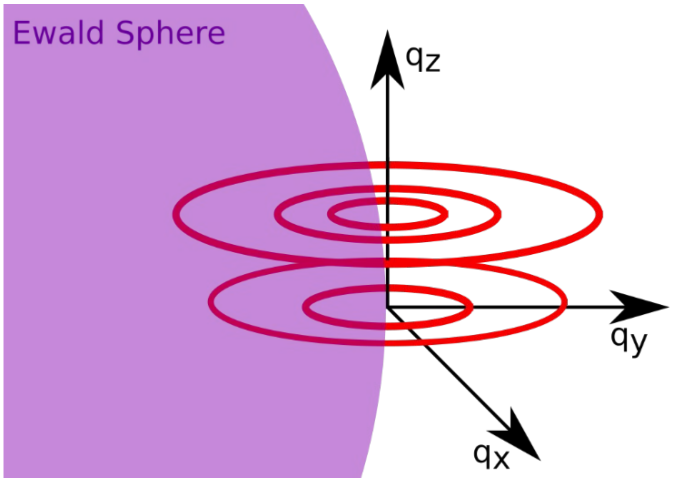

Supplementary Fig. 6 | Schematic representation of reciprocal space diffraction geometry for a two-dimensional powder. Intersections of the ring-shaped arrangement of the Bragg reflections with the Ewald sphere (shown in purple) manifest as diffraction peaks on the two-dimensional X-ray detector. In contrast, scattering from a three-dimensional powder (corresponding to spheres in reciprocal space) would result in powder rings on the detector.

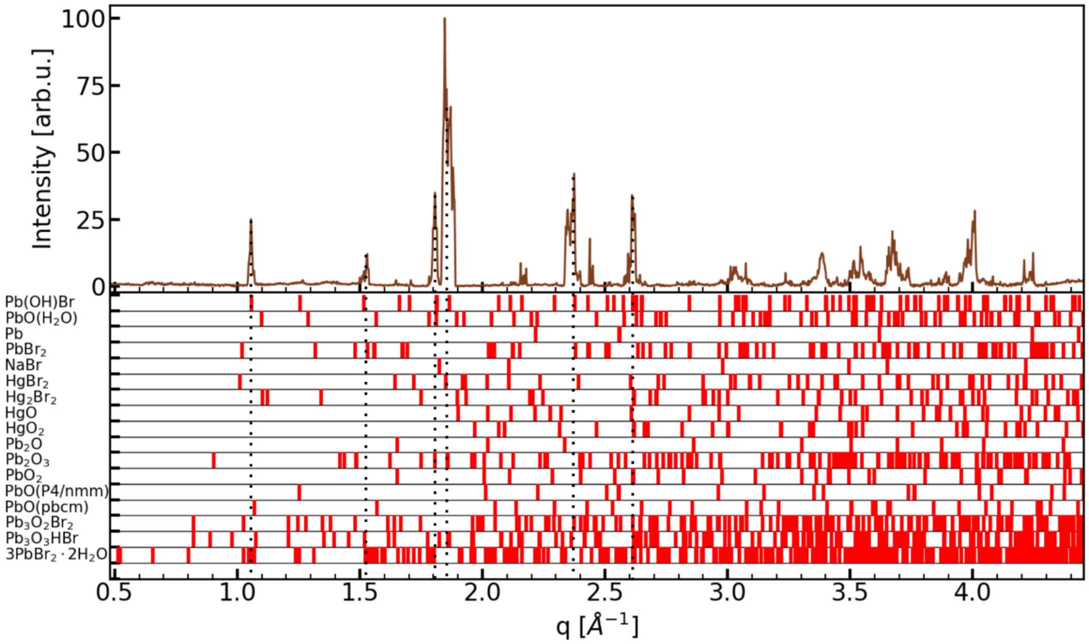

Supplementary Fig. 7 | Powder diffraction data of the bulk deposit formed in Br-containing electrolyte. Powder diffraction pattern obtained by integration of the GID data of the deposit in Br-containing electrolyte (top), compared to the peak positions of listed crystal structures (bottom).

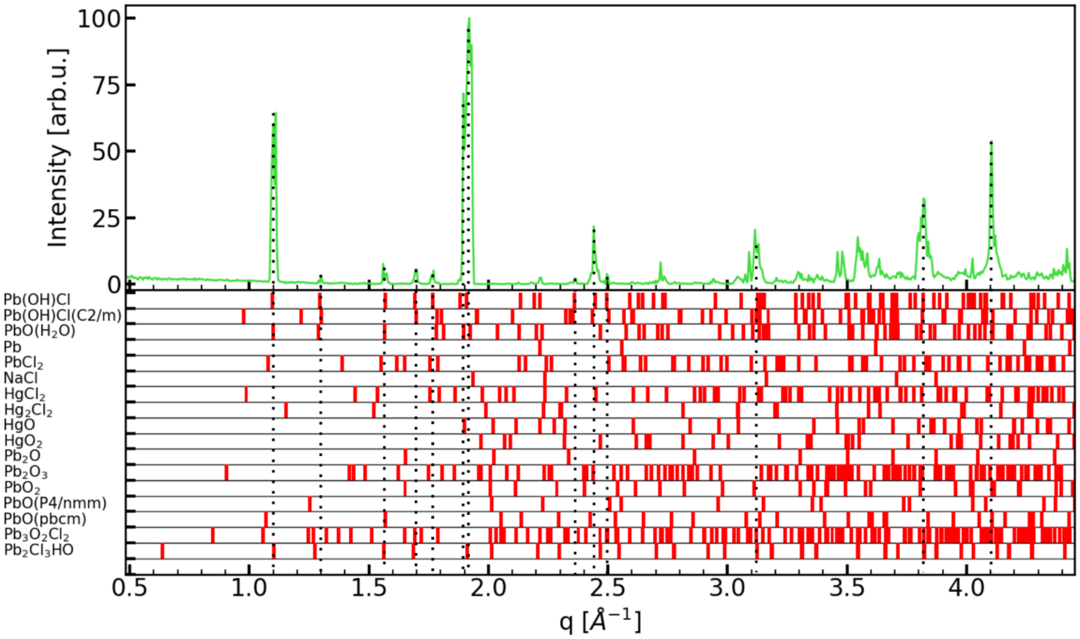

Supplementary Fig. 8 | Powder diffraction data of the bulk deposit formed in Cl-containing electrolyte. Powder diffraction pattern obtained by integration of the GID data of the deposit in Cl-containing electrolyte (top), compared to the peak positions of listed crystal structures (bottom).

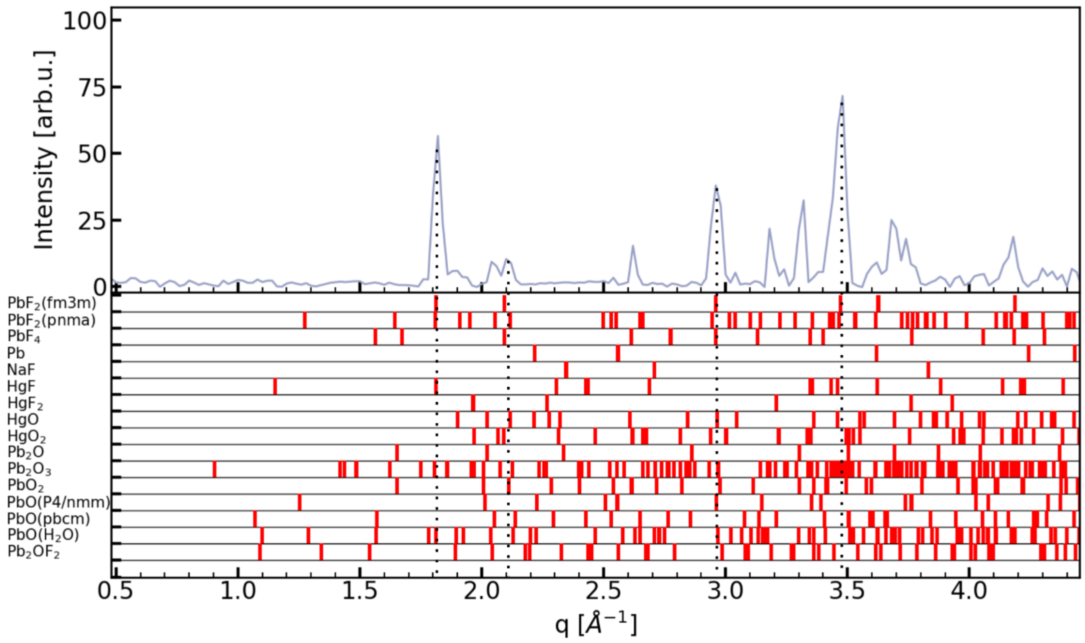

Supplementary Fig. 9 | Powder diffraction data of the bulk deposit formed in F-containing electrolyte. Powder diffraction pattern obtained by integration of the GID data of the deposit in F-containing electrolyte (top), compared to the peak positions of listed crystal structures (bottom).

Supplementary Methods 2: XRR modelling of the adlayer in Pb-containing solutions

This section describes the different models used in the fitting of the experimental data. For each system, we first considered an adlayer with a height of one full unit cell of the identified bulk material, as this was the adlayer structure found in the case of PbFBr[5]. That is, c-axis oriented Pb(OH)Br was used for Br-containing solution, c-axis oriented Pb(OH)Cl for Cl-containing solution, and PbF_2 for F-containing solution. Then we removed subsequently single Pb atomic layers (and their neighboring anion layers). For each of the XRR fits shown below (Supplementary Fig. 7, 9, 10), the solid line is the best fit described in the main manuscript and identical to the fit displayed in Fig. 2. The dashed line describes the best fit if only the majority phase $Pb(OH)X_{ad\text{-}I}$ (X = Br, Cl) or only two Pb layers (X = F) are considered.

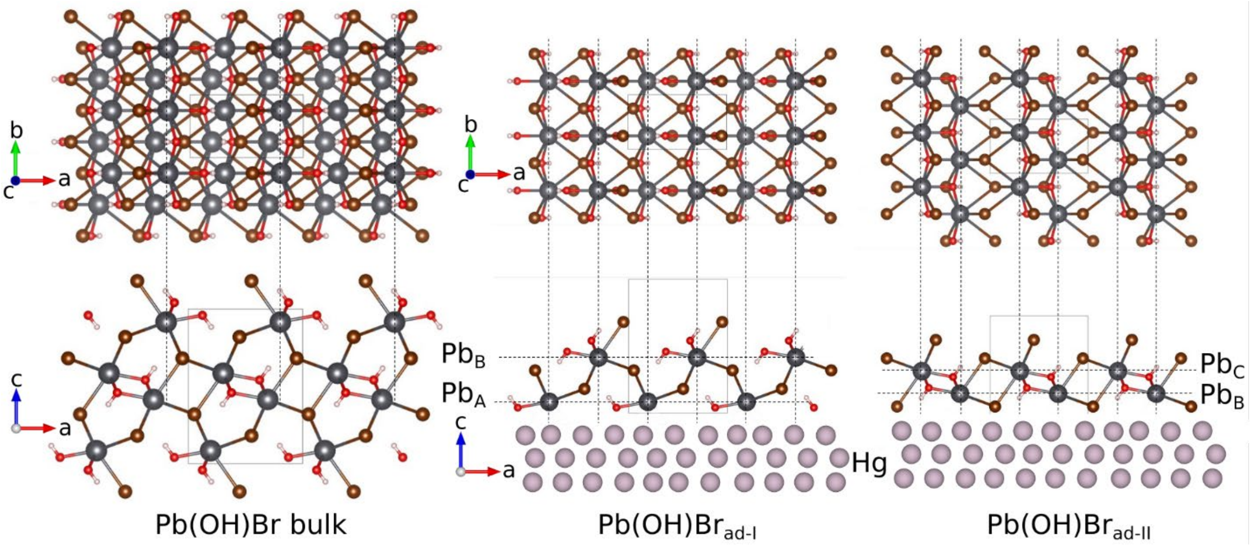

Supplementary Fig. 10 | Top and side view of the Pb(OH)Br crystal structure. On the left hand side, the bulk structure is shown, on the right hand side, the precursor adlayer structures used to fit the experimental XRR data at -0.80 V (generated by VESTA [6]).

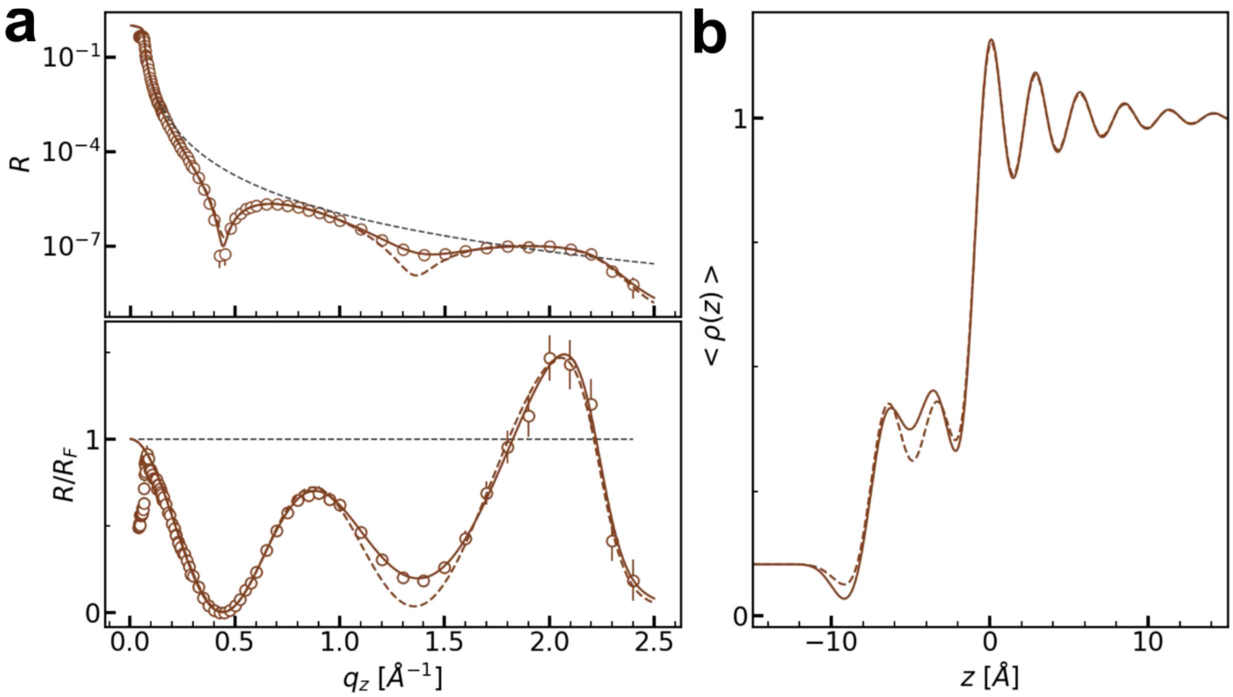

Supplementary Fig. 11 | Comparison of different models for describing the XRR data in Br-containing electrolyte. a X-ray reflectivity (top) and XRR normalized to the Fresnel reflectivity (bottom) of Hg electrodes in 0.01 M NaBr + 0.25 mM $PbBr_2$. The solid line describes the best fit by a model that includes two adlayer phases, $Pb(OH)Br_{ad}$-I and $Pb(OH)Br_{ad}$-II, the dashed line the best fit by a model with only $Pb(OH)Br_{ad}$-I. Obviously, fits with a $Pb(OH)Br_{ad}$-I adlayer phase alone cannot fully reproduce the oscillations in the XRR data, making the consideration of a second phase necessary. **b** Electron density profiles corresponding to the fits in **a**. The error bars represent the instrumental errors, resulting from the counting statistics of signal and background.

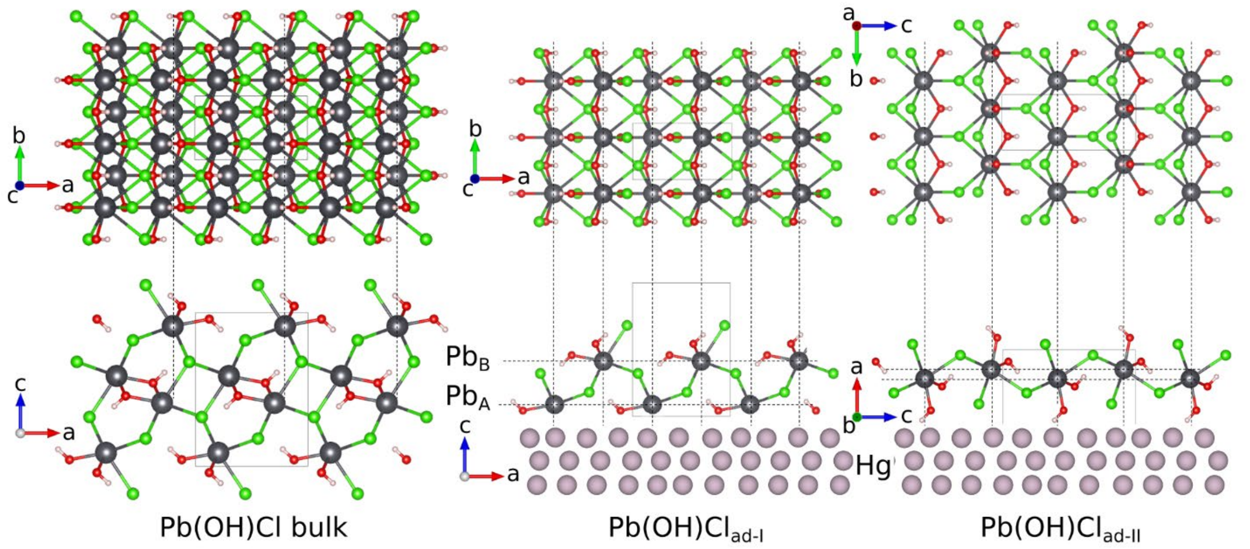

Supplementary Fig. 12 | Top and side view of the Pb(OH)Cl crystal structure. On the left hand side, the bulk structure is shown, on the right hand side, the precursor adlayer structures used to fit the experimental XRR data at -0.80 V (generated by VESTA [6]).

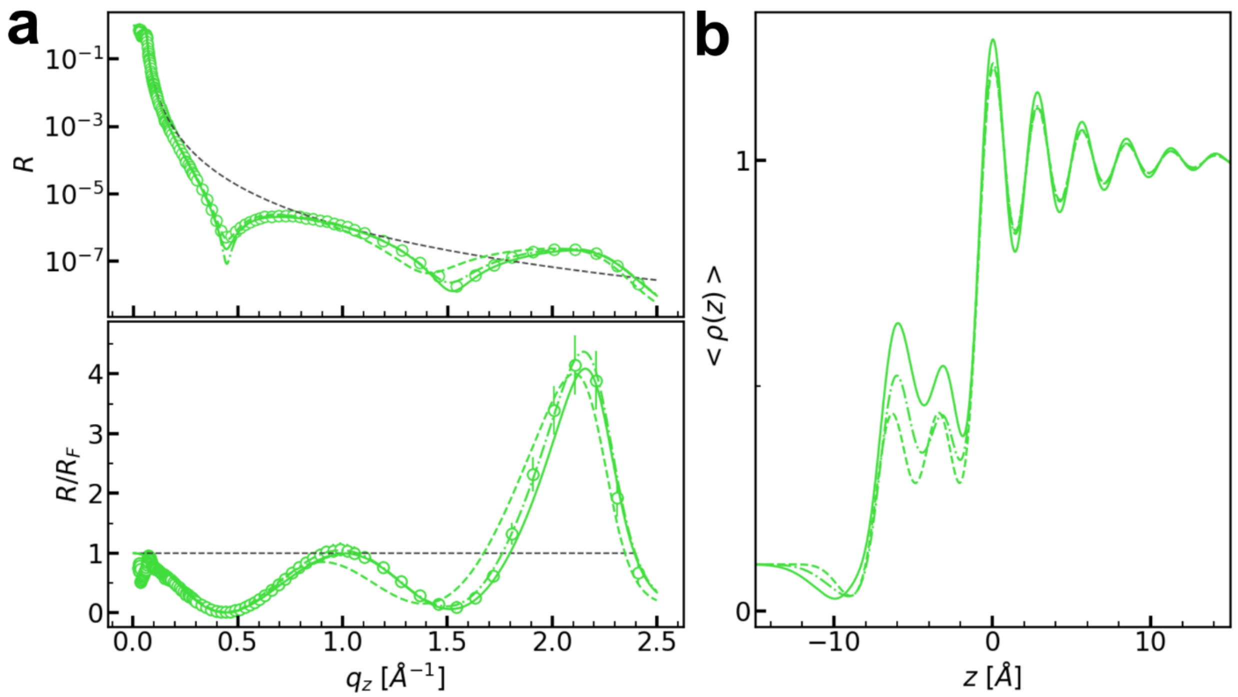

Supplementary Fig. 13 | Comparison of different models for describing the XRR data in Cl-containing electrolyte. a X-ray reflectivity (top) and XRR normalized to the Fresnel reflectivity (bottom) of Hg electrodes in 0.01 M NaCl + 0.25 mM $PbCl_2$. The solid line describes the best fit by a model that includes $Pb(OH)Cl_{ad\text{-}I}$ and $Pb(OH)Cl_{ad\text{-}II}$, the dashed line by a model with only $Pb(OH)Cl_{ad\text{-}I}$, and the dashed-dotted line by a model with $Pb(OH)Cl_{ad\text{-}I}$ and a c-axis oriented minority phase analogue to $Pb(OH)Br_{ad}$-II (but an unphysically large Cl-Hg distance of ~4 Å). Obviously, the first model provides the best description of the experimental data. **b** Electron density profiles corresponding to the fits in **a**. The error bars represent the instrumental errors, resulting from the counting statistics of signal and background.

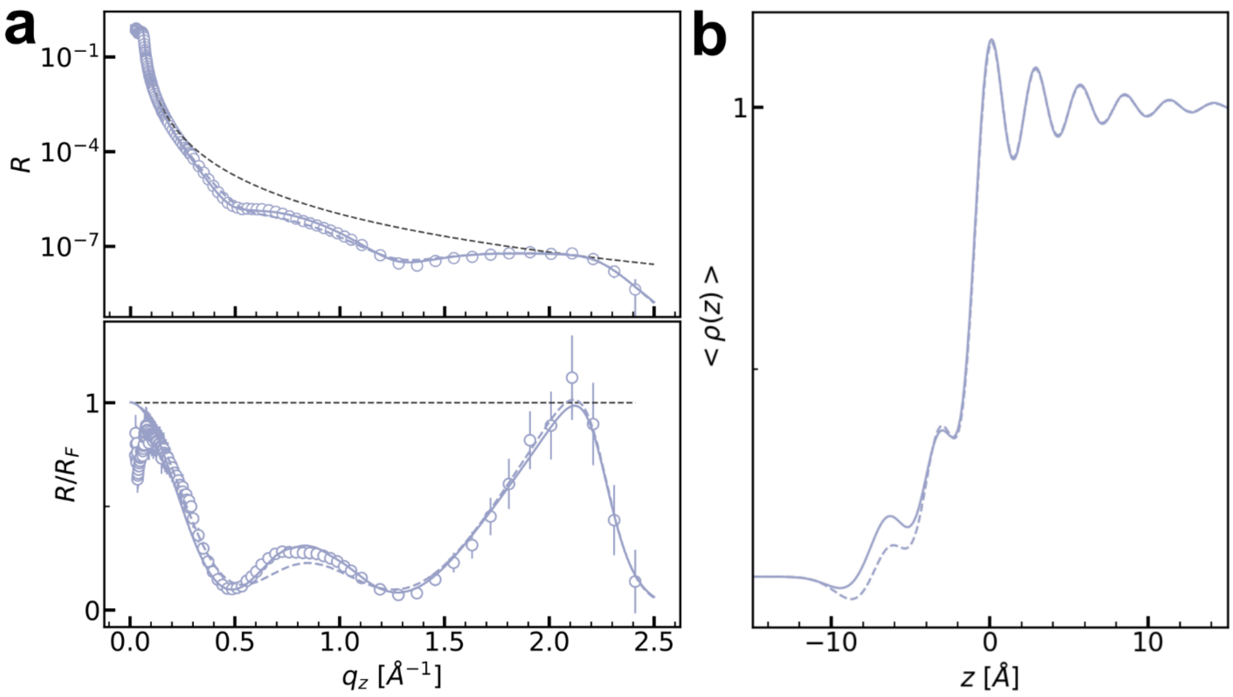

Supplementary Fig. 14 | Comparison of different models for describing the XRR data in F-containing electrolyte. a X-ray reflectivity (top) and XRR normalized to the Fresnel reflectivity (bottom) of Hg electrodes in 0.01 M NaF + 0.12 mM PbF_2. The solid line describes the best fit by a model including three Gaussians, representing Pb atomic layers, the dashed line by a model with two Gaussians. **b** Electron density profiles corresponding to the fits in **a**. The error bars represent the instrumental errors, resulting from the counting statistics of signal and background.

Supplementary Methods 3: Electrochemical cell for in situ XRR and GID studies

A custom-made electrochemical cell was used to collected the *in situ* XRR and GID data presented in the manuscript. The cell is made of PEEK and gas tight. Two opposite thin sections (0.5 mm) of the PEEK wall act as x-ray windows. Liquid Hg and electrolyte were inserted via connected PTFE tubings. A similar tubing connected a glass chamber with the reference electrode to the cell. The counter electrode was a ring of 0.5 mm thick Pt-wire along the inner circumference of the cell in order to have a homogeneous electric field at the working electrode. The electrolyte exchange was performed by applying an overpressure to the electrolyte reservoir, the replaced electrolyte was collected in a waste container. The flow was controlled by a series of valves. The microscope camera was mounted on top of the cell, where a quartz glass window provided an optical access. The cell also allows for temperature control but all measurements in this work were done at room temperature. A sketch of the electrochemical cell is shown in Supplementary Fig. 15.

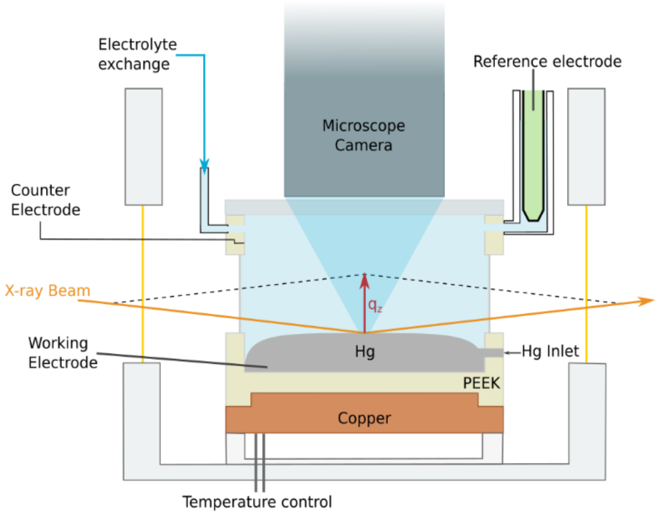

Supplementary Fig. 15 | Sketch of the electrochemical cell used for the experiment.

References:

1. Magnussen, O. M. *et al.* X-Ray Reflectivity Measurements of Surface Layering in Liquid Mercury. *Physical Review Letters* **74**, 4444–4447 (1995).

2. DiMasi, E., Tostmann, H., Ocko, B. M., Pershan, P. S. & Deutsch, M. X-ray reflectivity study of temperature-dependent surface layering in liquid Hg. *Physical Review B* **58**, R13419–R13422 (1998).

3. Elsen, A. *et al.* Surface Layering at the Mercury-Electrolyte Interface. *Physical Review Letters* **104**, 105501 (2010).

4. Runge, B. *et al.* Temperature- and potential-dependent structure of the mercury-electrolyte interface. *Physical Review B* **93**, 165408 (2016).

5. Elsen, A. *et al.* In situ X-ray studies of adlayer-induced crystal nucleation at the liquid–liquid interface. *Proceedings of the National Academy of Sciences* **110**, 6663 (2013).

6. Momma, K. & Izumi, F. VESTA 3 for three-dimensional visualization of crystal, volumetric and morphology data. *Journal of Applied Crystallography* **44**, 1272–1276 (2011).
